# Allergic conjunctivitis and the most common allergens in Northern Greece

**DOI:** 10.1186/1939-4551-6-12

**Published:** 2013-07-16

**Authors:** Diamantis Almaliotis, Pavlos Michailopoulos, Dimitrios Gioulekas, Paschalina Giouleka, Despina Papakosta, Thomas Siempis, Vasileios Karampatakis

**Affiliations:** 1 Pulmonary Department of the Aristotle, University of Thessaloniki, G. Papanikolaou Hospital, Eksohi, Chortiatis, Thessaloniki 57010, Greece; 2 Laboratory of Experimental Opthalmology, Medical School, Aristotle University of Thessaloniki, University Campus, Thessaloniki 54124, Greece; 3 Ophthalmolgy Department of G. Papanikolaou Hospital, Eksohi, Chortiatis, Thessaloniki 57010, Greece

**Keywords:** Allergens, Prevalence, Sensitization, Allergic conjunctivitis, Allergic rhinitis, Asthma, Skin prick tests

## Abstract

**Background:**

Ocular allergies affect a great part of the general population and often co exist with other allergic manifestations. In the present study, the prevalence of allergic conjunctivitis and the commonest allergens in allergic patients at an outpatient unit in Thessaloniki, Greece were evaluated.

**Methods:**

This is a retrospective study regarding allergic patients who referred to an outpatient clinic between the 1st of January of 1996 and the 31st of December 2010. They completed relative questionnaires concerning their allergic condition. The patients who were included in our study had allergic conjunctivitis confirmed by ophthalmologists and were divided into 4 groups. The criteria used were the existence of allergic conjunctivitis alone or with other allergic co- morbidities. The patients then underwent skin prick tests after consent according to current guidelines.

**Results:**

The archives of 1239 allergic patients were evaluated and 497 patients (40,11%) who manifested eye allergic symptoms were included in our study. 448 patients (90.14%) had allergic conjunctivitis in conjunction with asthma or rhinitis or both. 370 patients underwent skin prick tests and 284 of them (124 males-160 females) were positive for at least 1 of the 8 tested allergens (76.75%). 166 were positive to a grass mix (58.45%), 130 to olea European (45.77%), 124 to dust mites mix (43.66%), 58 to cypress (20.42%), 71 to parietaria officinalis (25.00%), 67 to cat dander (23.59%), 35 to dog dander (12.32%) and 32 to Altenaria (11.26%).

**Conclusions:**

Symptoms of ocular allergy are very common in patients with allergic rhinitis and asthma. Men had slightly higher percentages of positive skin prick tests, except for dog dander and Altenaria. Conjunctivitis should not be overlooked as an allergic entity when evaluating allergic patients.

## Background

Allergic conjunctivitis is commonly referred along with allergic rhinitis as “allergic rhinoconjunctivitis” due to the high-frequency of co-existence with allergic rhinitis and allergic asthma [1-6]. Despite the fact that allergic patients often present with other manifestations such as rhinitis, asthma, urticaria or eczema, ocular symptoms may be the initial and the most prominent indication of the allergic response.

In general, it is estimated that ocular allergies affect 5-22% of the population [7]. Especially in the USA, ocular allergy is estimated to affect 15-20% of the general population. The eye is a common site and target for the development of an allergic inflammatory disorder, in spite of the fact that tears may prevent the impact of allergens, such as pollens, on its surface [8]. Red eye is the most common sign of allergic conjunctivitis. Other common symptoms are watery eyes (88%), itching (88%), redness (78%), soreness (75%), swelling (72%) or stinging (65%) [9].

Ocular allergy, as an inflammatory disorder, consists of variable allergic manifestations with different presentations such as: a) seasonal allergic conjunctivitis (SAC), which is the most common one, b) perennial allergic conjunctivitis (PAC), c) giant papillary conjunctivitis (GPC), d) vernal keratoconjunctivitis (VKC) and e) atopic keratoconjunctivitis (AKC) [10]. GPC is usually associated with the use of contact lenses or caused by physical trauma. The most common types of ocular allergy are SAC and PAC [9]. AKC and VKC are characterized by chronic immune inflammation with T cell infiltration and may be sight threatening. On the contrary, SAC and PAC remain self-limited [11].

In practice, approximately 6% of consultations of general practitioners concern inflamed or red eye, half of which are caused by ocular allergy [12]. However, the allergic background of conjunctivitis is usually overlooked. Hence, allergic conjunctivitis is often under diagnosed and consequently under treated except when it is severe and the chief complaint of a consultation in a specialty clinic. Pharmacological treatment mainly includes the prescription of topical ocular mast cell stabilizers or antihistamines and in more severe cases corticosteroids, immunosuppressant drugs and immunotherapy [13,14]. The evaluation of the patients with skin prick tests (SPTs) is usually overlooked. SPTs represent an immediate IgE mediated allergic reaction and may provide clear evidence for the diagnosis of every specific allergic manifestation [1,15,16].

The aforementioned facts justify the need to identify the prevalence of allergic conjunctivitis in allergic populations and the common co existence of allergic conjunctivitis with other allergic manifestations, thereby enabling doctors to manage ocular allergies more effectively. In addition, the identification of the proportional frequencies of sensitization to the most common allergens provides a useful insight to exacerbating factors of allergic conjunctivitis.

The aim of the present study was to identify the prevalence of allergic conjunctivitis alone or in conjunction with allergic rhinitis and asthma and report the results of SPTs in an allergic population of Northern Greece.

## Materials and methods

This is a retrospective study regarding adult allergic patients who referred to an outpatient clinic between the 1st of January of 1996 and the 31st of December 2010. They completed relative questionnaires concerning their allergic condition which included 200 questions, needed approximately 15 minutes to fulfill and were performed with the assistance of a nurse. The abovementioned questionnaire is in use by the Pulmonary department of the Aristotle University of Thessaloniki for the last 30 years and provides information with regard to allergic symptoms (wheeze, dyspnoea, cough, sputum, rhinoroea, sneezing, tearing eye-itching, red eye), allergic background as well as previous medical diagnoses and therapies. The screening question for allergic conjunctivitis referred to watery, red and itching eyes.

Following the questionnaire for each entity (asthma, allergic rhinitis, allergic conjunctivitis) the diagnosis was confirmed by a specialist. The patients who were included in our study had allergic conjunctivitis confirmed by ophthalmologists and were divided into 4 groups. The criteria used were the existence of allergic conjunctivitis alone or with other allergic co- morbidities. The patients then underwent SPTs after consent, as required by the European Academy of Allergology and Clinical Immunology and the US Joint Council of Allergy Asthma and Immunology [17-19]. Positive SPTs for any of the 40 used allergens in each patient group were recorded. In addition, positive SPTs for the eight most common allergens according to international literature [grass mix, olive European, Parietaria officinalis, cypress, dust mites mix, cat and dog dander and Altenaria] were assessed for each patient. The allergen extracts were from the same manufacturer (Allergopharma-Germany). The SPTs were considered positive when the wheal diameter was ≥ 3 mm and redness ≥ 10 mm, 15 minutes after the test. The patients were instructed to avoid per os or topical use of antihistaminic drugs or steroids as well as anxiolytics [20].

## Results

1239 allergic patients (518 males – 721 females) referred to the clinic and completed the aforementioned questionnaires. 497 patients (186 males-311 females) aged 18 to 70 years old (mean age: 42.30 for males and 41.35 for females) had eye symptoms (40.11%) and were categorized in the following groups:

Group 1: 49 out of 497 (9.86%) patients had only conjunctivitis (C).

Group 2: 102 out of 497 (20.53%) patients had asthma and conjunctivitis (A + C).

Group 3: 117 out of 497 (23.54%) patients had rhinitis and conjunctivitis (R + C).

Group 4: 229 out of 497 (46.07%) patients had all three co-morbidities (conjunctivitis, asthma and rhinitis) (A + R + C).

370 out of 497 patients with allergic conjunctivitis underwent SPTs (127 patients refused to undergo SPTs due to social-economical reasons). 284 patients (124 males - 160 females) had positive SPTs for at least 1 of the 8 most common allergens (76.76%).

The prevalence of sensitization to the commonest 8 allergens (grass mix, olive European, parietaria officinalis, cypress, dust mites mix, cat and dog dander and Altenaria) and the relative frequencies are presented in Table 1.

**Table 1:** Prevalence of the commonest allergens and their proportional frequencies in 284 patients with allergic conjunctivitis

**Allergens**	**Number of examined patients**	**Positive SPTs (%)**	**Number of examined males**	**Positive SPTs (%)**	**Number of examined females**	**Positive SPTs (%)**
Grass mix	284	166 (58.45%)	124	78 (62.90%)	160	88 (55.00%)
Olive European	284	130 (45.77%)	124	61 (49.20%)	160	69 (43.10%)
Dust mites mix	284	124 (43.66%)	124	58 (46.80%)	160	66 (41.30%)
Cypress	284	58 (20.42%)	124	30 (24.20%)	160	28 (17.60%)
Parietaria officinalis	284	71 (25.00%)	124	35 (28.20%)	160	36 (22.60%)
Cat	284	67 (23.59%)	124	33 (26.60%)	160	34 (21.30%)
Dog	284	35 (12.32%)	124	15 (12.10%)	160	20 (12.50%)
Altenaria	284	32 (11.26%)	124	13 (10.50%)	160	19 (11.90%)

The foregoing results demonstrated that the most frequent allergens were grass mix 166 (78 males, 88 females), olive European 130 (61 males, 69 females) and dust mites mix 124 (58 males, 66 females).

Men had slightly higher percentages of positive SPTs for the majority of the allergens than women, except for dog dander and Altenaria to which women had a slight preponderance.

Moreover, it is worth mentioning that sensitization to seasonal allergens such as grass mix, Olive European, cypress and parietaria officinalis ,which may also induce pollen allergies, was very common. As far as the non- seasonal allergens are concerned the dust mites mix was the most frequent. On the contrary, sensitization to other non-seasonal allergens such as cat and dog dander as well Altenaria was lower. The results demonstrate that the prevalence of seasonal allergens is more frequent than perennial allergens except for dust.

In the present study, patients from group 2, group 3 and group 4, who had conjunctivitis in conjunction with at least one co-morbidity, were 448 (20.53% + 23.54% + 46.07%), while patients who had only allergic conjunctivitis were 49 (9.86%). Thus, 90.14% of patients had allergic conjunctivitis in conjunction with asthma or rhinitis or both. It is obvious then that allergic conjunctivitis may be overlooked since patients refer to the specialists for the most “noisy” symptoms of their allergy.

## Discussion

The present study evaluated patients who referred to an outpatient clinic unit and provides an insight regarding the prevalence of allergic conjunctivitis , either as an isolated entity or in conjunction with respiratory allergies, as well as the proportional frequencies of sensitization to the 8 commonest responsible allergens.

497 (40.11%) out of the 1239 examined allergic patients manifested allergic conjunctivitis. In this group, allergic conjunctivitis, without any other allergic manifestation, was detected only in 9.86% of the patients. Additionally, out of the 370 patients who underwent SPTs, 76.76% were positive for at least 1 out of the 8 allergens examined.

It is surprising then that there are not enough details in the international literature in regard to the prevalence of allergic conjunctivitis either as an isolated entity or as an allergic co-morbidity.

In one study that took place in the USA from 1988 to 1994, a specific questionnaire was used concerning ocular and nasal allergy symptoms in conjunction with SPTs [21]. The total number of patients was 20010. 1285 (6.4%) of them reported ocular symptoms, 3294 (16.5%) nasal symptoms, 5944 (29.7%) both ocular and nasal symptoms, and 9487 (47.4%) no symptoms at all. 40% of the cohort reported at least one occurrence of ocular symptoms in the past year. At the age of 50 years and older, the frequency of isolated ocular symptoms was higher due to the increase of dry eye symptoms in this age group. Nevertheless, in younger patients (up to 50 years old), there was an increase in the frequency of nasal symptoms both as an isolated manifestation and in combination with of ocular symptoms. Ocular symptoms in comparison to nasal symptoms were more frequent regarding sensitization to animals, household dust, and pollen [21].

In the present study, ocular symptoms and allergic conjunctivitis were more common in the younger ages. Moreover, our results indicated higher values of sensitization to pollen and household dust, but lower to animals. This maybe due to the fact that cat and dog dander are not seasonal allergens.

Raukas- Kivioja et. al in their study demonstrated that the prevalence of allergic conjunctivitis or rhinitis was 34.50% and the prevalence of positive SPTs was inversely related with the age. The authors also reported that sensitization to pollen was significantly associated with allergic conjunctivitis [22]. In our study sensitization to pollen was also high.

In another study by Navvaro et al. in a sample of 4991 patients, who referred for the first time in allergy medical services in Spain, 55% were diagnosed as having allergic rhinitis, 65% of whom had also conjunctivitis, and 37% asthma [23]. Pollen was the most frequent allergen (51%) followed by dust mites (42%). In our cohort the most frequent allergens, with positive SPTs, were grass mix (58.45%), olive European (45.77%) and dust mites mix (43.66%).

Wuthrich et al. evaluated the prevalence and severity of symptoms in 509 symptomatic Swiss patients with hay fever [24]. Conjunctivitis was confirmed in 93.3% of the cases (without any other allergic manifestations only in 8%), rhinitis in 92% (as an isolated entity in 6.7%), and asthma in 24.2%. The rhinitis group presented the most severe symptoms and the rhinoconjunctivitis group the least severe ones. In younger ages, conjunctivitis was more frequent than rhinitis, while asthma increased with age [24]. In our study, allergic conjunctivitis was confirmed in 90.14% of the cases and isolated conjunctivitis was diagnosed in 9.86% of patients, which indicates similar prevalence of conjunctivitis in these two studies.

Last but not least, in a population study in Helsinki by Pallasaho et al., family history of conjunctivitis or rhinitis was found to be a significant risk factor for allergic sensitization as well as the other pollens tested [25]. In our study family history was not taken into consideration. Our results are in agreement with those of Pallasaho et al., where males were at higher risks for presenting allergic symptoms than females, for any pollen and animal [25]. Moreover, in the same study it is indicated that living in urban areas in childhood until the first 5 years is associated with a greater risk to any pollen.

Even though the population in our study was not categorized in accordance to the areas where they lived (urban or countryside areas), the important message is that allergic conjunctivitis is usually hidden in the symptomatology of the respiratory allergies and is often wrongly considered as a common entity with rhinitis (rhino-conjunctivitis). As a result, the allergic background of conjunctivitis is frequently overlooked and physicians prescribe drugs without performing skin prick tests.

## Conclusions

The present study highlights that the frequency of allergic conjunctivitis is great in the allergic population and thus, ophthalmologists should play an important role in the management of the disease. The signs, symptoms and consideration of the background of allergic conjunctivitis as well as the therapeutic procedures should be assessed with the assistance of SPTs. It is interesting to outpoint that patients with allergic rhinitis and asthma may refer to allergy clinics and not to ophthalmologists.

On balance, symptoms of ocular allergy are very common among patients with allergic rhinitis and asthma. Allergic conjunctivitis, without any other allergic co-morbidity, was detected only in 9.86%. Hence, it should be kept in mind as an separate entity or co-morbidity with other allergies in the evaluation of allergic patients. The use of SPTs as a diagnostic tool provide useful information about the responsible allergen and enables us to instruct and cure patients more effectively.

## Abbreviations

SAC: Seasonal allergic conjunctivitis; PAC: Perennial allergic conjunctivitis; GPC: Giant papillary conjunctivitis; VKC: Vernal keratoconjunctivitis; AKC: Atopic keratoconjunctivitis; SPT: Skin prick test.

## Competing interests

The authors declare no competing interests.

## Authors’ contributions

AD conceived and designed the study, performed the statistical analysis and drafted the manuscript. PM analyzed the data, conducted the literature review and wrote part of the manuscript. DG collected the data, associated the results with the literature references and drafted the manuscript. PG collected the data, interpreted the results and wrote part of the manuscript. DP helped in the design of the study, the interpretation of the results and wrote part of the manuscript. TS: interpreted the data, wrote part of the manuscript and and extensively revised it. VC: overlooked and coordinated the study as well as drafted and revised the manuscript. All authors have given final approval of the version to be published.
